# Validation of automated artificial intelligence segmentation of optical coherence tomography images

**DOI:** 10.1371/journal.pone.0220063

**Published:** 2019-08-16

**Authors:** Peter M. Maloca, Aaron Y. Lee, Emanuel R. de Carvalho, Mali Okada, Katrin Fasler, Irene Leung, Beat Hörmann, Pascal Kaiser, Susanne Suter, Pascal W. Hasler, Javier Zarranz-Ventura, Catherine Egan, Tjebo F. C. Heeren, Konstantinos Balaskas, Adnan Tufail, Hendrik P. N. Scholl

**Affiliations:** 1 Institute of Molecular and Clinical Ophthalmology Basel (IOB), Basel, Switzerland; 2 OCTlab, Department of Ophthalmology, University Hospital Basel, Basel, Switzerland; 3 Department of Ophthalmology, University of Basel, Basel, Switzerland; 4 Moorfields Eye Hospital NHS Foundation Trust, London, United Kingdom; 5 Department of Ophthalmology, Puget Sound Veteran Affairs, Seattle, Washington, United States of America; 6 eScience Institute, University of Washington, Seattle, Washington, United States of America; 7 Department of Ophthalmology, University of Washington, Seattle, Washington, United States of America; 8 Royal Victorian Eye and Ear Hospital, Melbourne, Victoria, Australia; 9 Moorfields Ophthalmic Reading Centre, London, United Kingdom; 10 Supercomputing Systems, Zurich, Switzerland; 11 Institut Clínic d’Oftalmologia, Hospital Clínic de Barcelona, Barcelona, Spain; 12 Institute of Ophthalmology, University College London, London, United Kingdom; 13 Wilmer Eye Institute, Johns Hopkins University, Baltimore, Maryland, United States of America; Politechnika Krakowska im Tadeusza Kosciuszki, POLAND

## Abstract

**Purpose:**

To benchmark the human and machine performance of spectral-domain (SD) and swept-source (SS) optical coherence tomography (OCT) image segmentation, i.e., pixel-wise classification, for the compartments vitreous, retina, choroid, sclera.

**Methods:**

A convolutional neural network (CNN) was trained on OCT B-scan images annotated by a senior ground truth expert retina specialist to segment the posterior eye compartments. Independent benchmark data sets (30 SDOCT and 30 SSOCT) were manually segmented by three classes of graders with varying levels of ophthalmic proficiencies. Nine graders contributed to benchmark an additional 60 images in three consecutive runs. Inter-human and intra-human class agreement was measured and compared to the CNN results.

**Results:**

The CNN training data consisted of a total of 6210 manually segmented images derived from 2070 B-scans (1046 SDOCT and 1024 SSOCT; 630 C-Scans). The CNN segmentation revealed a high agreement with all grader groups. For all compartments and groups, the mean Intersection over Union (IOU) score of CNN compartmentalization versus group graders’ compartmentalization was higher than the mean score for intra-grader group comparison.

**Conclusion:**

The proposed deep learning segmentation algorithm (CNN) for automated eye compartment segmentation in OCT B-scans (SDOCT and SSOCT) is on par with manual segmentations by human graders.

## Introduction

Optical coherence tomography (OCT) is one of the most rapidly evolving imaging technologies used in ophthalmology to visualize eye structures [1, 2]. Due to the growth in available imaging datasets and increased computing powers, novel automated pattern recognition methods have been proposed to detect retinal pathologies such as age-related macular degeneration or presence of macular fluid [3, 4].

Until recently, methods for automated image analysis relied on hand-crafted rule sets or classical machine learning. These include, for example, path search using gradient information [5], classification of image patches using kernel regression [6], or random forest classification [7].

In recent years, deep learning with artificial multi-layer neural networks has become very successful in visual learning tasks [8, 9]. In particular, convolutional neural networks (CNN) have been shown to outperform other computer vision algorithms [10, 11]. For semantic segmentation tasks, i.e., pixel-wise labelling, various neural network architectures have been proposed [12, 13]. In particular, the so-called U-Net architecture has proven to be among the most reliable and performant semantic labelling algorithms in medical imaging applications [14, 15]. This is because of its characteristic U-shape, which enables it to combine semantic information describing what is shown in an image with spatial information.

Within the field of ophthalmology, deep learning has been successfully applied [16, 17, 18] to detect macular edema [19, 20], for retinal layer segmentation [21], or to determine retinal thickness [22].

In this paper, we build upon U-Net for the purpose of OCT image compartment segmentation. Most U-Net applications strongly rely on the original implementation [23]. In this paper, we increased the field of view with an additional layer in depth as opposed to introducing residual blocks as previously reported [24]. This allowed us to consider larger spatial regions of the input images to classify individual pixels.

In contrast to automated image analysis, major limitations of manual image segmentation by human graders are its time-consuming nature, training of expert-level graders, variable reproducibility, and the need for relatively high-quality images compared to automated measurements [25].

The purpose of this study was to propose and benchmark a deep-learning-based algorithm to segment OCT B-scans. To our knowledge, this is the first study to comprehensively compare a deep learning algorithm against different levels of human graders on both SDOCT and SSOCT images. This was especially important, as there is a growing use of non-domain experts as graders (e.g., crowd sourcing) or for training novice retina/eye doctors/employees [26, 27].

Specifically, our novel contributions are:

- We propose the first retina compartmentalization tool using a dual CNN approach made feasible for both types of OCT image acquisition techniques (SDOCT B-and SSOCT scans).
- Our trained CNN is the first to detect the choroid compartment in its entirety on complete B-scans to calculate new parameters such as surface area and volume.
- To the best of our knowledge, our presented study is based on a higher number and diversity of manual human graders that make it possible to study both the intra- and inter-observer variability among human graders, and to measure the human grader’s performance against the trained CNN’s performance.
- The data obtained will not only be valuable from a technical point of view, but will also lead to values that are of great clinical significance in upcoming longitudinal studies—for example, those on pigmented tumors of the choroid.
- The developed method will be further enhanced and made available as a fully automated and cloud-based analysis method for pigmented choroidal tumors free of charge and open access to the medical community.

## Material and methods

The use of the data for this study was pre-approved by the local ethics committee Nordwest- und Zentralschweiz (EKNZ) (ID: EKNZ UBE-15/89 and EKNZ UBE-l5/72) and was performed in accordance with the tenets of the Declaration of Helsinki. Written informed consent was obtained from all subjects.

### Data

The inclusion criteria were: complete display of all three compartments (vitreous, retina, choroid), good represented retina layers with three out of three possible values in the acquisition of SSOCT, and image quality of at least 25 in SDOCT imaging, indicated by the OCT device. Exclusion criteria were known vitreous or retinal surgery, retinal pathologies such as retinal scars, gliosis, traction, incomplete retina scans, and blinking artifacts.

The data set consisted of 2130 B-scans from 630 healthy OCT C-scans of both spectral-domain OCT (SDOCT) (1076 B-scans: scan length 6 mm, 384x496, 1024x496 and 768x496 pixels, enhanced depth imaging off, averaged for 25 scans using the automatic averaging and tracking feature), and swept-source OCT (SSOCT) (1054 B-scans: scan length 6mm, 512x992 pixels).

The OCT data were obtained from Heidelberg Spectralis HRA+ OCT (Heidelberg Engineering, Heidelberg, Germany), and swept-source OCT from DRI-OCT-1 Atlantis DRI (Topcon Inc., Tokyo, Japan).

### Manual annotation of B-scans

**Fig 1:**
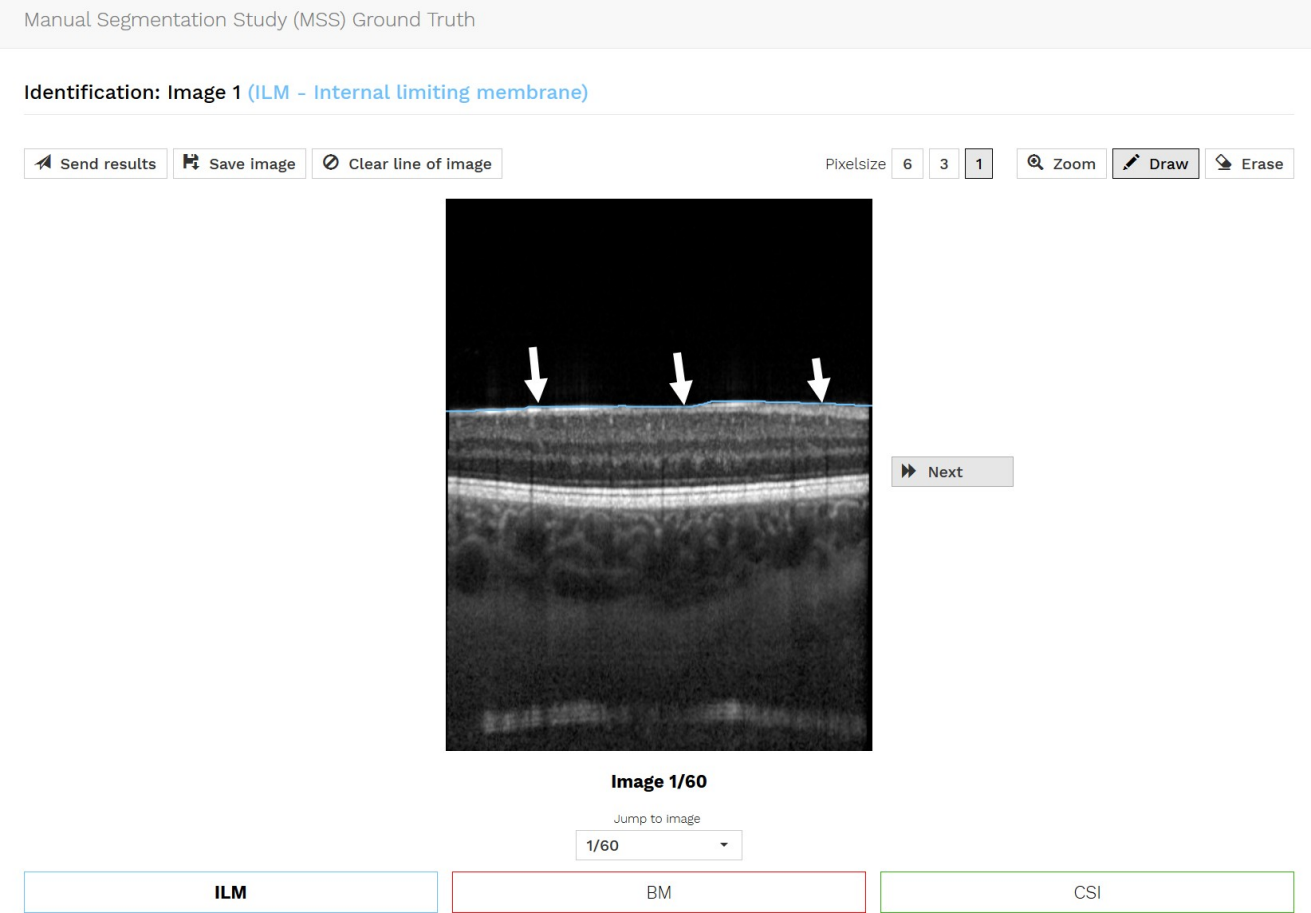
Illustration of the online annotation tool used for manual OCT image compartmentalization. In this example, the ILM (arrows) was segmented. The same was done for inner border of the choriocapillaris (CC) and CSI.

The manual annotation of the ground truth for the four eye compartments was conducted by independent human graders using an individual and password protected online tool specifically developed for OCT B-scan image annotation (Fig 1). After logging in to the grading tool, the grader was asked to draw three lines corresponding to the demarcation line between the hyporeflective vitreous and well-reflective inner retinal boundary marked as “internal limiting membrane” (ILM), the internal delineation of the choriocapillaris (CC), and the choroid-sclera interface (CSI). The vitreous was defined above the ILM reaching until the top of the image border; the retina was defined from the ILM to and including the inner border of the CC. This landmark was chosen because Bruch's membrane is normally too thin and is not recognizable in OCT as a separate line and the choriocapillaris appears dark in conventional images due the flow. The choroid was defined below the CC to and with the CSI; and the sclera was defined below the CSI until the lower image border.

The resulting segmentation lines and the segmentation times for each line were stored. Based on these acquired lines, the compartments of each OCT-image were computed, resulting in pixel-wise labels stored as images. These generated label images contain four types of labels: vitreous, retina, choroid, and sclera.

All the graders used the same 20-page tutorial explaining the annotation of the required lines.

The whole data set of 2130 B-scans was annotated with regard to the three lines once by one ground truth expert ophthalmologist (GT EO). Sixty of those B-scans were also annotated as a benchmark by nine human graders: 1) three OCT naïves, 2) three expert ophthalmologists (EO), and 3) three members of a reading center (RC). For the OCT-naïves or laymen (L), non-medical subjects were included who had no prior knowledge of OCT imaging. The expert ophthalmologists were three medical retina fellows from the Moorfields Eye Hospital, London, UK. Finally, three experts from the Moorfields Eye Hospital Reading Centre were included. In total, nine human graders manually annotated 60 benchmark B-scan images three times each to test for reproducibility of the method. After annotating the benchmark B-scans for the first time, they were randomly shuffled for the second and third annotation runs.

### Ground truth

2070 of the OCT B-scans annotated by the GT EO were augmented by mirroring each image once and randomly rotating each image once between -8 and 8 degrees resulting in a data set of 6210 OCT B-scan images. This set was then split into a training set of 4968 images and a validation set of 1242 images. The training data set was used to train the CNN, and the validation set was used to determine when training should stop and for the hyper-parameter selection.

Finally, the benchmark data set, which was annotated by nine human graders as well as the GT EO, consists of 30 SDOCT and 30 SSOCT images. This data set was used to benchmark the performance of the CNN and compare it to the human graders as well as the variability of the human graders. With this comparison, we demonstrate that the compartmentalizations of the proposed algorithm lie within the range of the variability within and among human graders.

The ground truth data sets are summarized in Table 1.

**Table 1:** Overview of the three ground truth data sets.

Ground truth	# Images	Augmented	# Graders
Training data set	4968	Yes	1
Validation data set	1242	Yes	1
Benchmark data set	60	No	9+1

### Comparison of compartmentalizations

For benchmarking the OCT image compartmentalization, the segmentation outputs were compared to each other by calculating the pixel-based IOU or Jaccard [28] score of the compartments (vitreous, retina, choroid, and sclera):

$$IOU=\frac{Intersection}{Union}$$

The closer the score is to 1, the higher is the assessed overlap/agreement.

We assessed the results of our proposed algorithm and investigated the human grader variability by comparing 1) the predictive performance of the CNN with respect to the GT EO, from which the CNN learned, 2) the predictive performance of the CNN with respect to the three grader groups, 3) the intra-grader variability, and 4) the inter-grader variability. The comparisons are based on the 60 B-scans of the benchmark data set. Box plots are used to summarize the results of the respective comparisons.

### Statistical analyses

We performed statistical tests to investigate the significance of the difference of 1) the variabilities among human graders, and 2) the variabilities between human graders and the CNN. When IOU scores were approximately normally distributed, we performed two-sided, unpaired t-tests assuming different variances in the two sets of IOU scores being compared. When IOU scores were not normally distributed, we performed unpaired Mann-Whitney U-tests.

### CNN architecture

**Fig 2:**
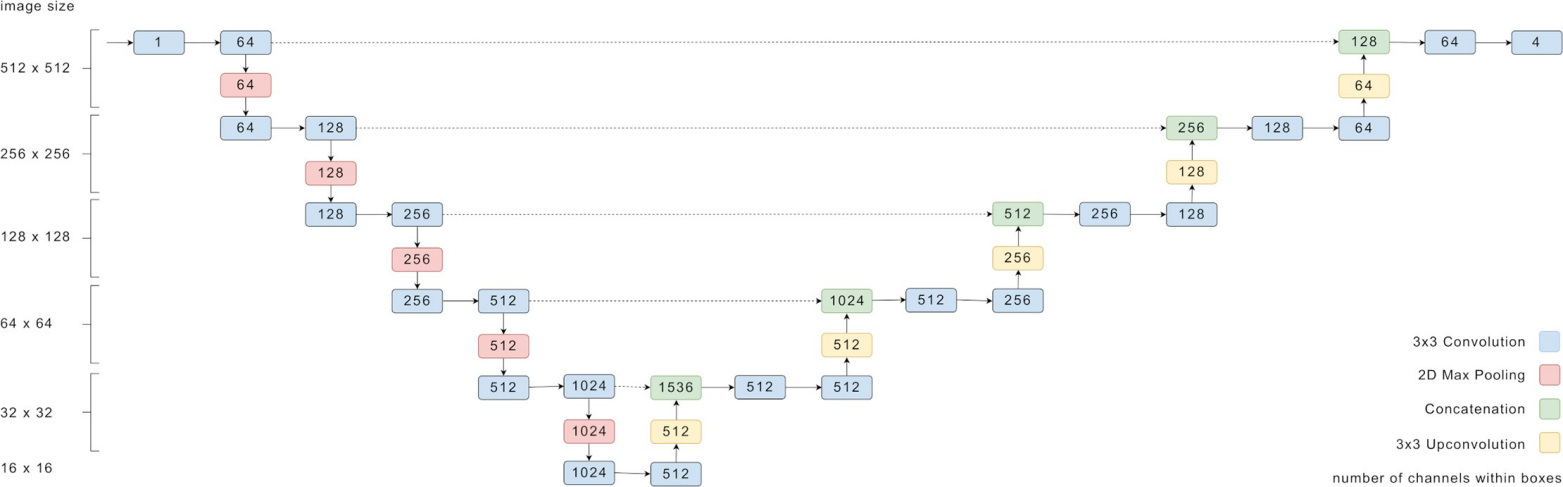
Overview of the U-net structure of the CNN used in this work. The numbers denote the dimensions of tensors passed between the layers.

For the compartment segmentation, we trained a CNN based on U-Net [29], which was implemented using the TensorFlow framework [30]. The CNN architecture is illustrated in Fig 2. The main extension to the original U-Net is the additional layer in depth that allows for images of 512 by 512 pixels to be completely covered by the field-of-view of the network. The OCT ground truth used for the CNN training was resized to 512x512 pixel images using bicubic interpolation for the images and nearest neighbor interpolation for the segmentation masks. No further preprocessing was applied.

### CNN training and predictions

The CNN was trained using an unweighted pixel-wise cross-entropy loss and the weights were adjusted using an Adam optimizer [31, 32]. The weights were initialized with the Xavier method. A mini-batch size of four images, and a learning rate of 6e-5 were used. The neural network was trained for 10 epochs on a single NVIDIA GTX TITAN X GPU. The training took approximately three hours and was kept short to avoid overfitting. No further regularization methods were applied.

For predictions, the CNN output is passed through a softmax function and each pixel is classified according to the highest probability.

## Results

The graders’ mean ophthalmic training time was 0, 6.67 (from 6 to 7), and 9.3 (from 6 to 15) years for OCT naïves, expert ophthalmologists (EO), and reading center experts (RC), respectively. The ground truth grader had 21 years’ ophthalmic expertise.

In this section, we compared the predictive performance of the CNN to the GT EO, from which the CNN learned to segment OCT B-scans. Furthermore, we described the intra-grader variability based on the three manual segmentations, which we obtained from each grader for each of the 60 B-scans of the benchmark data set. We then compared the CNN-generated segmentations to the manual segmentations of each of the three grader groups (RC, EO, L). Finally, we provided the inter-grader variability within each grader group and showed that the average difference between a CNN-generated segmentation and a manual segmentation is similar to the average difference between two manual segmentations from any two graders of the same grader group.

### CNN versus GT EO

**Fig 3:**
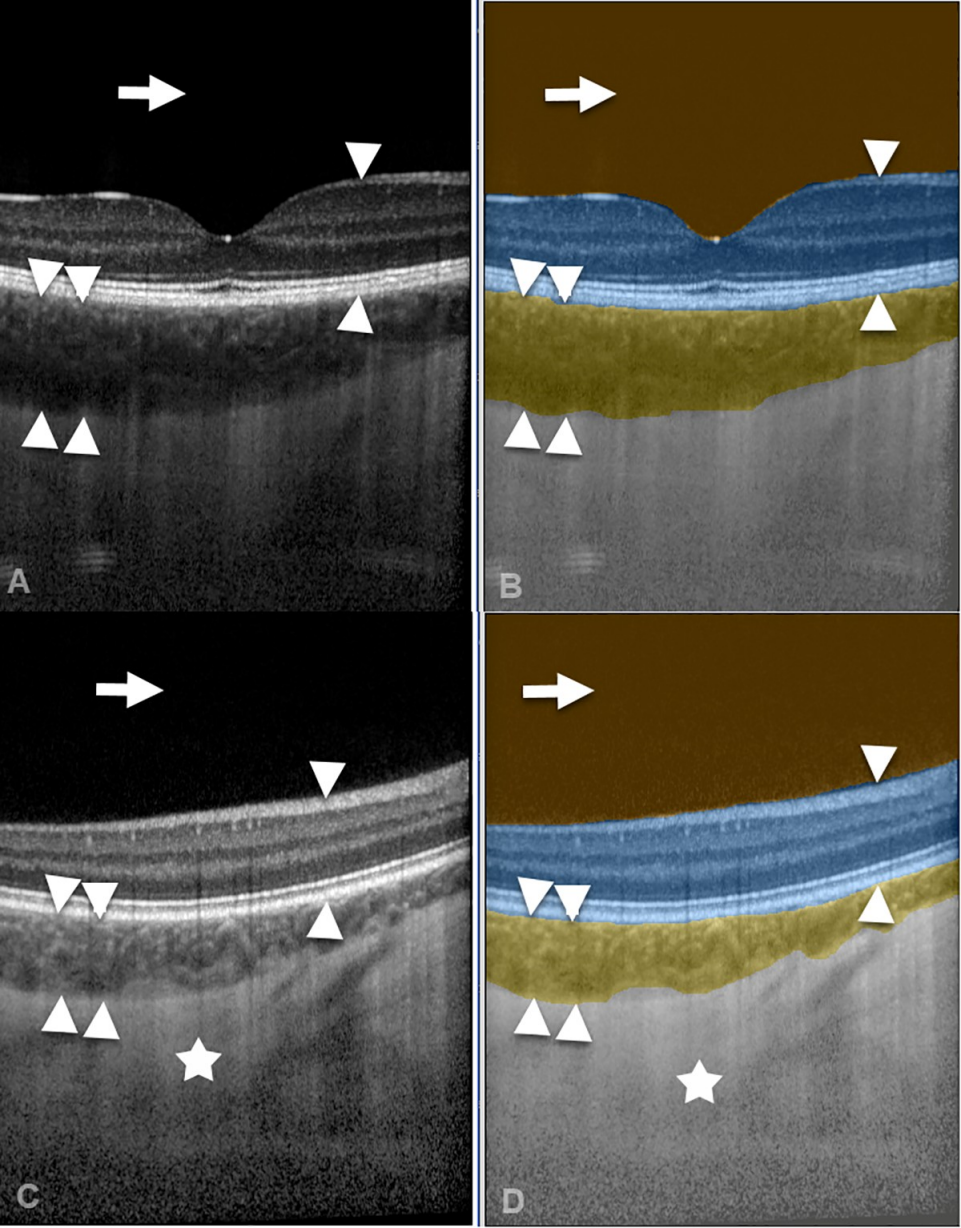
Illustration of automated artificial intelligence OCT image compartmentalization. A spectral-domain OCT image (A) and a swept-source OCT image (C) were automatically segmented by the CNN (B, D) into the compartments vitreous (arrow), retina (arrow heads), choroid (double arrow heads), and sclera (asterisk).

Examples of automated CNN compartmentalization of OCT images are depicted in Fig 3. The CNN compartmentalization achieved average IOU scores of 0.9929 for vitreous, 0.9690 for retina, 0.8817 for choroid, and 0.9768 for sclera when compared to the 60 benchmark images labeled by the GT EO.

### Intra-grader analysis

**Fig 4:**
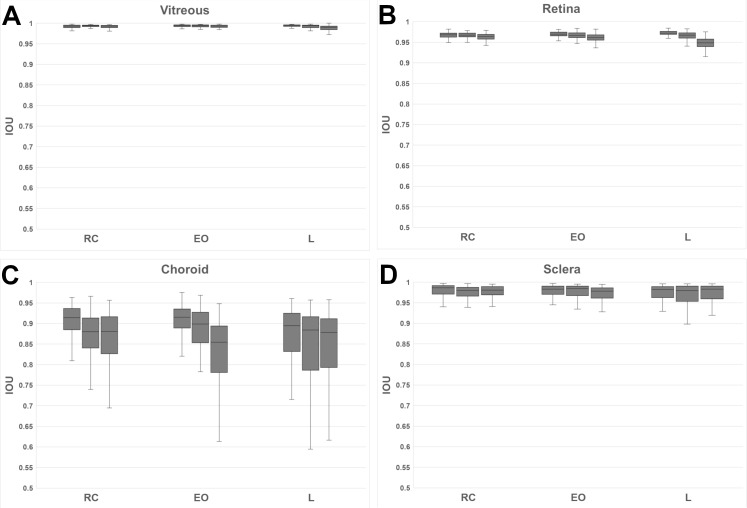
Intra-grader variability for each grader group per compartment class:(A) vitreous, (B) retina, (C) choroid, and for the (D) sclera.

The variability of every single grader was assessed by calculating the aforementioned IOU scores for all three possible comparisons between the three runs of a grader. Specifically, the images from run 1 were compared to runs 2 and 3 and run 2 was compared to run 3. This results in 180 IOU scores per grader and per compartment. Fig 4 shows box plots of the resulting scores for each grader per compartment class. The graders have been grouped by their respective group RC, EO, and L.

The scores reflect how consistently each grader annotated the images. In general, the scores for all graders are very high for vitreous and high for retina and sclera. Hence there is strong agreement among annotation runs. The variability for vitreous and retina is low and moderate for sclera. The choroid compartment depicts most variability and the lowest scores, in particular for the L group. Both EO and RC groups show a higher consistency for choroid labels.

Choroid and sclera scores are both influenced by the graders CSI line which has, however, a stronger influence on the choroid score since the sclera has a larger compartment area.

### Inter-grader analysis

For the inter-grader analysis, we split the analysis into the three grader groups: RC, EO, and L. For each group we compared the first run of each grader to the first runs of the other group members. This results in 180 IOU scores per group. In contrast, the CNN predictions and the first runs of the three graders were compared, resulting in 180 scores, too.

**Fig 5:**
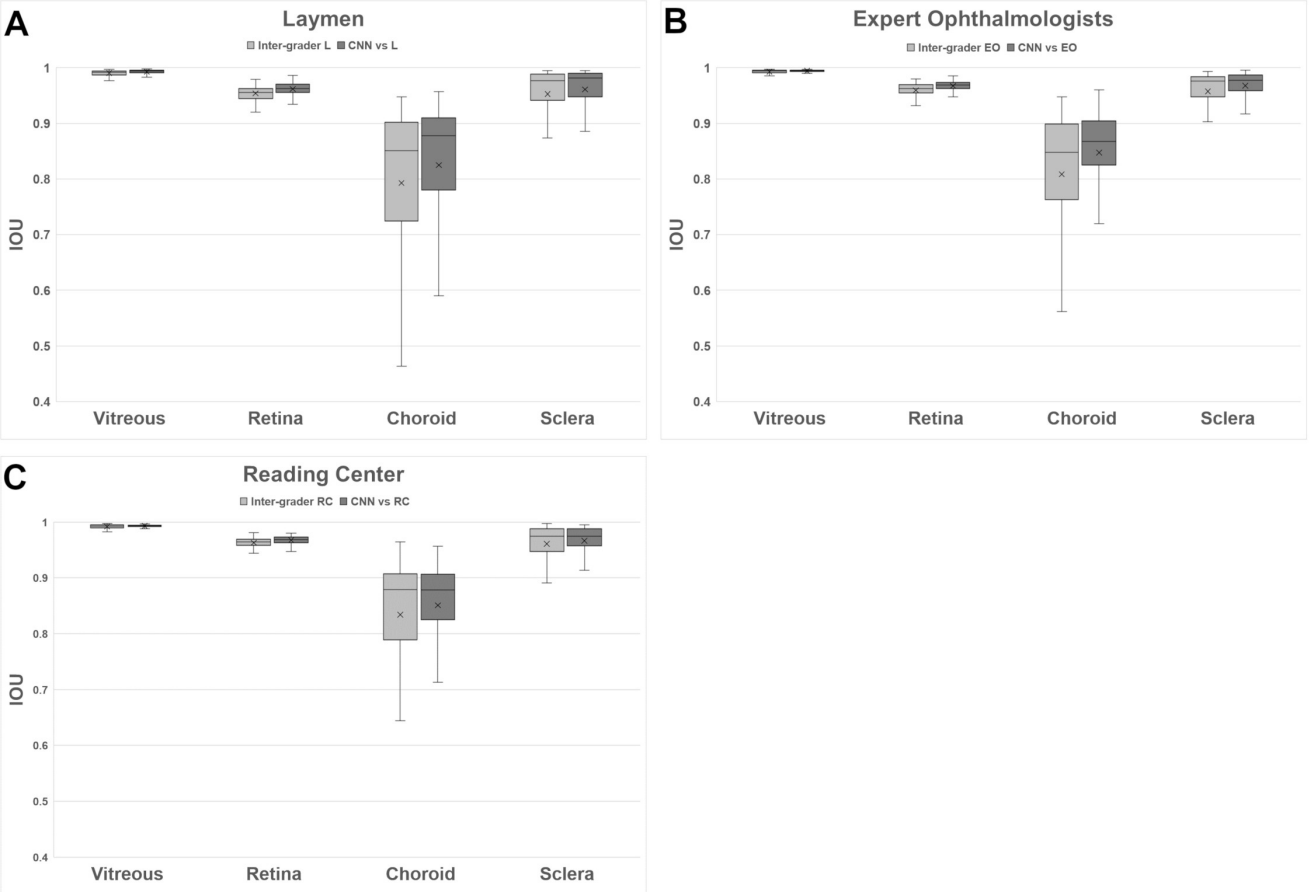
Inter-grader agreement within each group for (A) layman group (L), (B) expert ophthalmologist group (EO), and (C) reading center (RC) group, with regard to the compartments of (A) vitreous, (B) retina, (C) choroid, and (D) sclera. For each group, the predictions of the CNN were compared with the groups’ results.

The results are plotted in Fig 5. The vitreous and retina compartments show high scores and high agreement within each group. At the same time, the variability of scores of the CNN compared to the groups is lower and the average IOU higher. Two-sided t tests revealed that in each group the mean IOU score between the CNN and the graders was significantly higher than the mean inter-grader IOU score (Table 2).

**Table 2:** Results of statistical tests comparing inter-grader IOU scores and IOU scores between CNN and graders. Two-sided t tests were performed when data was approximately normally distributed. Non-parametric Man-Whitney U-tests were performed, when data was not normally distributed.

Group	Compartment	Test	p-value
L	Vitreous	Two-sided t test	1.151e-06*
L	Retina	Two-sided t test	1.95e-09*
L	Choroid	Man-Whitney U-test	0.02895
L	Sclera	Man-Whitney U-test	0.1701
EO	Vitreous	Two-sided t test	1.369e-06*
EO	Retina	Two-sided t test	1.219e-10*
EO	Choroid	Man-Whitney U-test	0.004588*
EO	Sclera	Man-Whitney U-test	0.02977
RC	Vitreous	Two-sided t test	0.000669*
RC	Retina	Two-sided t test	5.056e-07*
RC	Choroid	Man-Whitney U-test	0.65
RC	Sclera	Man-Whitney U-test	0.8525

Cross symbols * indicate statistically significant results at the 1% confidence level.

For the sclera compartment, a moderately higher variability in IOU scores were observed. In all groups, inter-grader IOU scores as well as IOU scores between CNN and graders did not appear to be normally distributed. Therefore, non-parametric Mann-Whitney U-tests were performed to investigate whether inter-grader IOU scores and IOU scores between CNN and graders originated from the same distribution. At the 1% significance level, the IOU scores were not statistically significantly different for the L and RC groups and statistically significant for the EO group (Table 2).

The greatest disagreement in IOU scores was found for the choroid compartment. Although the IOU scores were generally high for the choroid compartment, there was significant variability within each group of graders. The variability of choroid IOU scores is highest for Ls and lowest for RCs. Furthermore, a large difference between median IOU and lowest IOU is observed for choroid, indicating a strong disagreement in only some of the images. The choroid CNN variability is lower than the intra-group variability for all groups. Additionally, the choroid CNN scores including the median are higher for Ls and EOs and only slightly lower for RCs. However, Mann-Whitney U-tests did not reveal that inter-grader IOU scores and IOU scores between CNN and graders were statistically different (Table 2).

## Discussion

This study was intended to identify strengths and weaknesses of human and automated artificial intelligence (AI) annotation by means of compartmentalization of optical coherence tomography (OCT) images. The main goal was achieved by developing a neural network that showed to be at least as good as human graders. This is an important intermediate step in the development of a CNN that will measure pigmented choroidal tumors fully automatically in the future. Because this project was developed by the Institute of Molecular and Clinical Ophthalmology Basel (IOB), a research institute that combines basic and clinical research and is governed as a foundation, the proposed method will be shared with the community in a novel cloud solution at no charge.

Despite the great progress in AI image segmentation investigation, many ambiguities still remain as to how the technology functions in detail. There is incomplete understanding of the decision space of deep learning [33] and its layer depth architecture [34] that can potentially impair automated ophthalmic image analysis. Therefore, we benchmarked a hitherto unprecedented number of three classes and nine independent graders with two levels of ophthalmic proficiency (EO, L) to be compared to the Moorfield’s Reading Center (RC), which is specialized in credible, reproducible, and methodical analysis of ophthalmic images, and specifically to the recently developed CNN in order to find new evidence.

The study revealed interesting results regarding both the grader groups as well as the automated CNN segmentation results. The presented results are comparable to previous studies, although it should be noted that in our study the number of images and graders was much higher [35, 36]. The intra-grader analysis suggests that all groups segment for vitreous and retina with high consistency. Annotating the CSI, however, is the most challenging segmentation step, which is reflected in the lower IOU scores for choroid and sclera. The group-based inter-grader analysis confirmed the results of the intra-grader analysis: While the groups had a strong agreement for the vitreous and retina compartments, the groups scored lower and with higher deviation for the choroid and sclera compartments. The results of this study suggest that the manual segmentation of the CSI must be considered with the utmost caution, because they showed the largest deviations. Causes for the deviations can be that the choroidal layer looked to be very closely connected to the sclera tissue, without an in-between boundary being recognizable. In addition, a different interpretation as to whether choroidal tissue columns should be segmented or not could have influenced the manual segmentation results.

From this perspective, laymen image segmentation tasks performed during crowd ground truth generation for machine learning can be justified in some cases and subject to reservation, taking into account that the CSI showed the largest deviations. Indeed, expert segmentation such as from the Moorfield’s RCs will still result in higher quality segmentations, which can be favored in order to train a machine, which will then be utilized and shared by numerous researchers as we have planned in our open access case.

The automated segmentation approach using a trained CNN achieved—except for the RCs—a more consistent result for all compartments at high scores comparable to human graders. For vitreous and retina compartments, the CNN achieved a statistically significant higher average IOU score between CNN and graders compared to inter-grader IOU scores. In the case of choroid and sclera compartments, inter-grader IOU scores and IOU scores between CNN and graders were not statistically significantly different from each other, with the exception of the choroid compartment in the EO group. While it would be ideal to have the images segmented by RCs most of the time, it is known that the resources of the RC are limited and cannot be consulted for every single OCT B-scan. Therefore, the results of this study are important, since the CNN eliminates two of the greatest weaknesses of the human grader approach. Firstly, the CNN segmentation repeatedly produces the same segmentation for a given OCT B-scan, making segmentation results reproducible and independent from the graders’ experience of segmentation. Hence, the CNN sets a benchmark with a reliable segmentation quality for OCT image compartmentalization for the research and clinical community. In addition, such a compartmentalization benchmark can help to measure the initial performance of new graders and objectively quantify their progress after training units, which is very important for a RC to make the quality of the employed graders comparable and even compare to other reading centers.

Secondly, the CNN segmentation releases human graders from a cumbersome manual segmentation, which on the one hand is time consuming, but on the other hand also represents a tedious task demanding concentration and attention to detail. Furthermore, in contrast to conventional AI image analysis, alternative technologies such as transfer learning algorithms are expected to predict the subject class which can facilitate the information processing [37].

Limitations of the study are that in some cases the CSI border was not very clearly delineated because connective tissue columns were densely intergrown and therefore no sharp dividing line was visible. The amount of data is relatively small. However, the results are encouraging and should be further improved. The results were collected from healthy eyes and the performance of the algorithm in diseased eyes and other image scalings must be validated in upcoming studies.

### Conclusions and future work

This study demonstrates that a deep learning neural network can segment both SDOCT and SSOCT for the eye compartments vitreous, retina, choroid, and sclera. The CNN-based segmentation provided reliable and accurate compartmentalization of retinal layers comparable to that of human graders. This technology offers exciting potential for large-scale, high-quality OCT image compartmentalization for both research and patient monitoring purposes.

With this work, the feasibility of automated compartmentalization of retinal zones in healthy eyes was validated. Hence, with the help of this tool it is possible to reliably classify large numbers of OCT B-scan stacks in a reproducible automated way. This classified data can then be used to analyze and study 3D OCT images. This first intermediate step is an encouraging result to further promote the developed technology to also enable it to detect pathologies—for example, of pigmented choroidal tumors. For this purpose, a cloud-based platform has already been developed, which will enable remote machine learning OCT scan analysis for tumors to be researched, detected, segmented, and, above all, objectively quantified in 3D. This will lead to a democratization of the machine learning technology, which will be accessible to less privileged users. Ideally, the system will be fed by the community with a larger number of cases to continuously enhance the quality. Only anonymized data must be used, for which we already developed an OCT-specific tool that will be made available for open access.

## Supporting information

S1 Table Results for grading with regard to intergrader variability are included. (XLSX)

S2 Table Results for grading with regard to intragrader variability are included. (XLSX)
